# The Effect of Exercise Training Intensity on VO_2_max in Healthy Adults: An Overview of Systematic Reviews and Meta-Analyses

**DOI:** 10.1155/2022/9310710

**Published:** 2022-02-24

**Authors:** Emmet Crowley, Cormac Powell, Brian P. Carson, Robert W. Davies

**Affiliations:** ^1^Department of Physical Education and Sport Sciences, Faculty of Education and Health Sciences, University of Limerick, Limerick, Ireland; ^2^Physical Activity for Health Cluster, Health Research Institute, University of Limerick, Limerick, Ireland; ^3^High Performance Unit, Sport Ireland, Sport Ireland National Sports Campus, Dublin, Ireland

## Abstract

This study aimed to evaluate systematic reviews and meta-analyses that have examined the effect of exercise training on VO_2_max in healthy individuals at different intensities. Five databases were searched: EBSCOhost, MEDLINE/PubMed, SPORTDiscus, Web of Science, and Google Scholar. Eligibility criteria for selecting reviews included systematic reviews and meta-analyses of healthy adults that examined the effect of lower intensity training (LIT) and/or high intensity training (HIT) on VO_2_max. Eleven reviews met the eligibility criteria. All reviews were of moderate-to-very strong methodological quality. The included reviews reported data from 179 primary studies with an average of 23 ± 10 studies per review. All reviews included in this overview showed that exercise training robustly increased VO_2_max at all intensities. Three meta-analyses that compared LIT versus HIT protocols on VO_2_max reported small/moderate beneficial effects for HIT over LIT; however, the beneficial effects of HIT on VO_2_max appear to be moderated by training variables other than intensity (e.g., training impulse, interval length, training volume, and duration) and participants' baseline characteristics (e.g., age and fitness levels). Overall, evidence from this overview suggests that the apparent differences between LIT and HIT protocols on VO_2_max were either small, trivial, or inconclusive, with several methodological considerations required to standardise research designs and draw definitive conclusions.

## 1. Introduction

VO_2_max is the gold standard measure of cardiorespiratory fitness [1, 2] and a strong predictor of cardiovascular health, morbidity, and all-cause mortality [3–5]. Therefore, an improvement in VO_2_max (i.e., the functional limit of the cardiorespiratory system) can reduce the risk of cardiovascular disease and mortality—even when other risk factors are present (e.g., ageing, hypertension, diabetes, smoking, and obesity) [4, 6]. Exercise training is an effective means of increasing VO_2_max [1, 2]. Therefore, clear recommendations for exercise intensity are required to facilitate optimal and efficient improvements in cardiorespiratory fitness.

The prescription of training intensity falls into two broad categories: lower-intensity training (LIT) and higher-intensity training (HIT). A standardised approach to the categorisation of intensity has been frequently established (e.g., LIT refers to exercise bouts <80% VO_2_max, whereas HIT refers to exercise bouts >80% VO_2_max [7]). And within these categories, three exercise modalities are commonly prescribed within the literature: (1) moderate-intensity continuous training (MICT); (2) high-intensity interval training (HIIT); and (3) sprint interval training (SIT). MICT usually refers to training programmes consisting of extended duration continuous exercise at moderate intensities (e.g., 50–80% VO_2_max) [8, 9]. HIIT is a form of interval training, which refers to intermittent exercise that involves alternating higher intensity with lower intensity [10], with high intensity (e.g., 80–170% VO_2_max) bouts of exercise between 30 seconds and 4 minutes [11]. SIT is another form of interval training, which consists of maximal intensity (e.g., maximal exertion, >170% VO_2_max), but shorter durations, of up to 30 seconds [12, 13]. Both LIT (i.e., MICT) and HIT (i.e., HIIT and SIT) protocols have been shown to significantly improve VO_2_max in most populations (e.g., young, elderly, active/athletic, and sedentary) [14, 15]; however, which, or if any, exercise training intensity is most effective at increasing VO_2_max remains unclear.

There are several systematic reviews and meta-analyses available in the existing literature examining the effect of exercise training on VO_2_max. However, very little is known about the overlap of the primary studies included in these systematic reviews and/or meta-analyses. Hence, the different methodologies employed within each systematic review/meta-analysis will influence results (e.g., intensity standardisation and prescription, eligibility criteria, data analysis, etc.), making it difficult to draw definitive conclusions from any single review. Therefore, the primary aim of this paper was to perform an overview of systematic reviews and meta-analyses that have examined the effect of exercise training on VO2max, at different intensities, in healthy/nonclinical populations. In addition to an overview of the available evidence, our secondary aim was to provide practical applications based on findings and make key suggestions for future research for establishing evidence-based recommendations on exercise training intensity. In this regard, an overview of systematic reviews and meta-analyses provides an opportunity to map and summarise the evidence to date, highlight limitations in the extant literature, absence of evidence, and identify the key variables that may influence the effect(s) of exercise training intensity of VO_2_max.

## 2. Methods

### 2.1. Search Strategy

Electronic database searches were performed through EBSCOhost, MEDLINE, PubMed, SPORTDiscus, Web of Science, and Google Scholar using all available records up to 30 July, 2021. The literature search, quality assessment, and data extraction were conducted independently by two authors (EC and CP) and any discrepancies were resolved following discussion with a third author (RD). A combination of search terms was used (Table 1).

### 2.2. Study Criteria

This review had a series of inclusion and exclusion criteria, which were limited to systematic reviews and/or meta-analyses articles. The inclusion criteria were (1) exercise/training interventions; (2) randomised and nonrandomised controlled trials with intervention(s) on healthy adults; (3) exercise (intensity) group(s); (4) control group(s); (5) written in English only; and (6) distinction of data, for extraction, between experimental groups. The exclusion criteria were (1) no measure of VO_2_max; (2) cross-sectional study design; (3) patient groups; (4) strength training; (5) concurrent training; (6) nutritional interventions; and (7) masters, paraplegic, and/or athletes with clinical conditions.

### 2.3. Selection of Systematic Reviews and/or Meta-Analyses

**Figure 1:**
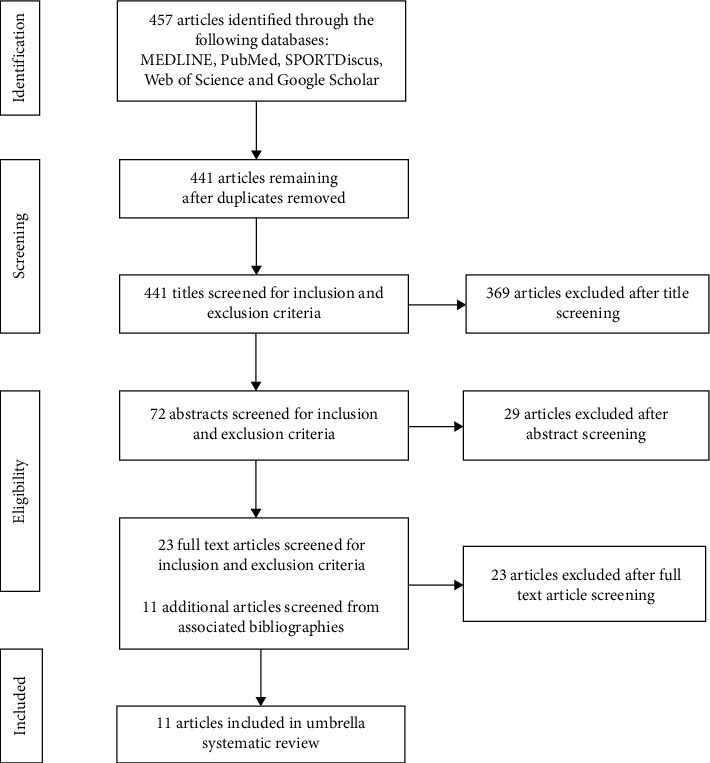
Schematic representation of the data extraction protocol. The PRISMA flowchart was used to illustrate the inclusion and exclusion criteria used in this overview.

Systematic reviews and meta-analyses were selected using the Preferred Reporting Items for Systematic Reviews and Meta-Analyses (PRISMA) guidelines (Figure 1) [16]. The search process included a hierarchy of assessment whereby papers were first assessed by journal title (and duplicates removed), second by abstract, and third by full-article review when the journal article was either included or excluded based on the eligibility criteria.

### 2.4. Summary Measures

Data were extracted from systematic reviews and meta-analyses under two key areas: background information and training interventions with VO_2_max as the outcome measure. Background information included (1) type of review/analysis (e.g., systematic review, meta-analysis, and metaregression); (2) the number of studies included in each review; (3) training intensity groups (i.e., control, MICT, HIIT, and SIT, which fall under the umbrella terms of LIT or HIT, resp.); (4) sample size in each training group, and (5) baseline characteristics (e.g., sex, age, and training status). The following training intervention information was also extracted: (1) modality of exercise (e.g., running, cycling, etc.); (2) duration of the intervention; (3) frequency of training (weekly); (4) exercise intensity (i.e., %max); and (5) change in VO_2_max or pre/post-training results. For accuracy of interpretation, all intensity descriptors (i.e., numerical and categorical) were extracted from their original reviews ad verbatim and were not recategorised within the results section of this current paper.

### 2.5. Study Quality Assessment

A Measurement Tool for the Assessment of Multiple Systematic Reviews (AMSTAR) checklist was used to rate the quality of the literature [17]. The tool consists of 11 items and has good face and content validity for measuring the methodological quality of systematic reviews [17]. The total quality score for each included review ranged from 0 to 11. The quality of the review was labelled as either weak (score range: 0–3), moderate (score range: 4–7), or strong (score range: 8–11).

If primary studies are included in more than one review, this can lead to bias (pseudoreplication) in the interpretation of the results. A citation matrix of the primary studies was constructed to assess the degree of overlap between the systematic reviews/meta-analyses included in the overview. The degree of overlap was assessed by the percentage of primary studies included in >1 systematic review/meta-analysis and the correct covered area (CCA): (*N–r*)*·*(*r · c–r*)^−1^, where *N* is the total number of studies (including double-counting); *c* = number of reviews; *r* = number of unique studies, indicating slight (0 to 5%), moderate (6 to 10%), high (11 to 15%), or very high (>15%) overlap [18].

## 3. Results

Table 2 includes details of the population characteristics of the 11 included reviews. The reviews included were systematic [15–18, 23, 24, 26], and/or meta-analyses [14, 15, 19, 21–27], and/or meta-regressions [22]. Taken together, the eleven included reviews covered a total of 179 primary studies at 23 ± 10 (range: 9 to 41) studies per review. Forty-nine (27%) of the primary studies were included in two or more reviews. The CCA was 4.7%, indicating a “slight” degree of overlap between systematic review/meta-analyses.

### 3.1. Study Characteristics

AMSTAR scores (Table 2) for the included reviews were either moderate [6, 7, 19, 21, 22, 25] strong [8, 9, 14, 15, 20, 23, 26, 27], or very strong [10, 24]. The number of studies included within each review ranged from 9 [24] to 41 [19]. Control groups included were either defined as: a nonexercise control group (CON) [19, 22] or an exercising control group (EX-CON) [14, 26], which nominally differed from MICT but was approximately the same intensity, and/or a MICT [14, 15, 19, 23, 24, 27] group, with HIIT [14, 21–25] or SIT [15, 26, 27] accompanying the EX-CON or CON groups. The reviews, in some instances, only reported the total sample size [21, 24, 25], with other reviews including the breakdown of participants across the training groups. All reviews included both male and female participants; however, it was evident that there was a greater number of male participants (68% male vs. 27% female), with 5% of reviews not differentiating between male and female participants. The age of the participants ranged from young healthy adults (>18 y) [14, 23, 26] to older healthy adults (>70 y) [19, 21].  Table 3 highlights the effect of different training methods on VO_2_max across the reviews. Primary modes of exercise included running [19–22, 26, 27] and cycling [14, 15, 19–22, 26, 27], with other modes of exercises reported, such as tai chi [19] and snowshoeing [22]. Duration of exercise training intervention(s) ranged from 4 to 38 weeks [15, 26]. Training frequency ranged between 2 and 5 times per week, with all reviews reporting a mean training frequency of 3 training sessions per week.

### 3.2. Intensity Prescription

**Figure 2:**
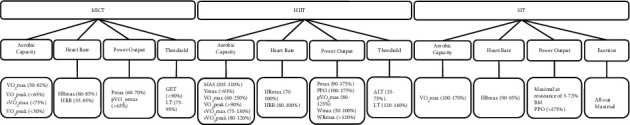
Flow chart diagram of exercise intensity prescription across the eligible reviews.

Training intensities (Table 3) prescribed ranged from LIT (60% VO_2_max or 70% HRmax) [23, 27] to supramaximal (“all out”) HIT [14, 15, 20, 23, 26, 27]. Exercise intensity was prescribed using a range of measures (Figure 2) other than VO2max [14, 15, 20, 23, 25, 27], including maximal heart rate (HRmax) [20, 24, 26, 27], heart rate reserve (HRR) [19–21, 23, 24, 26]), other VO_2_max variables (maximal velocity at VO_2_max (*V*_max_) [14, 19–24, 26], velocity at VO_2_max (vVO_2_max) [19–21, 23], gas exchange threshold (GET) [14, 20, 23], peak oxygen uptake (%VO_2_peak) [23], maximal aerobic power (pVO_2_max) [14, 15, 20, 26], maximal aerobic speed (MAS) [14, 26]), power output (peak watt work load (Pmax) [14], work rate at VO_2_max (%WRmax) [23], maximal power output (%Wattmax) [21, 24], peak power output (%PPO) [20, 26, 27]), lactate threshold variables (lactate threshold (%LT) [23, 27]; velocity at lactate threshold (%VLT) [23, 27], change between lactate thresholds VO_2_max (∆LT) [27]), and maximal exertion (“all out” [20, 23]). The measures reported in this overview were categorised in line with the training modality (i.e., MICT, HIIT, and SIT) and accompanied by the number of studies and range of intensities prescribed. It is important to note that the categorisation of intensity within HIT groups differs and is not in line with previously standardised categories [25]. For example, Montero et al. [21] classify HIT at VO_2_max between 60 and 80%. However, Montero et al. [24] in a younger population (22–28 years) prescribed HIT as a VO_2_max between 60 and 95%. Additionally, it is important to note that definitions of variables of power output differ between reviews, and in some instances, different definitions equate to the same prescription measure. For example, Pmax [14], %WRmax 19, %Wattmax [21, 24], and %PPO [20, 26, 27] provide similar measures of power output but use different terminology. Therefore, within Tables 2 and 3, intensities were categorised in accordance with the original reviews and not recategorised; however, these intensity variations were taken into consideration for the purpose of the discussion section. Finally, VO_2_max as an outcome measure was either reported as mL.kg.min^−1^, L.min^−1^ or % change in VO_2_max.

### 3.3. Exercise Outcome

All reviews included in this overview showed that exercise training increased VO_2_max (Table 3). Reviews that used a nonexercise control comparator showed a significant improvement in VO_2_max following LIT (i.e., MICT [14, 19]) and HIT (i.e., HIIT [14, 20, 22, 25] and SIT [20, 26]) [27]. Six meta-analyses directly compared HIT and LIT modalities [14, 20, 22, 23, 26], which allowed for a meta-analytical comparison between training intensity groups. Of these, three [20, 23, 27] reported small/moderate beneficial effects for HIT on VO_2_max over LIT [20, 23], but had a high degree of overlap between primary studies (CCA = 11%). However, it is important to highlight some discrepancies within/between the reviews included in this overview, which resulted in exclusion from the intensity comparison. For example, Montero et al. [21, 24] included both a HIIT (HIT group) and an MICT (LIT group) group; however, the intensities prescribed for HIIT were low (e.g., 60–95% VO_2_max) compared to other reviews included in this overview. These reviews could not be considered for further analyses as they did not have a distinctive HIT group. Therefore, the findings are principally here limited to a young healthy participants.

## 4. Discussion

### 4.1. Meta-Analysis Findings

Taken together, the eleven included reviews reported data from 179 unique primary studies. However, further investigation shows the limitation of current systematic reviews and/or meta-analyses, with an average of only 23 ± 10 studies per review, which highlights the need for this current overview. The findings of this overview show that LIT and HIT are both effective at increasing VO_2_max in both young and old, healthy, and sedentary, adults; however, some methodological considerations require attention on the interpretation of these findings. Evidence from the meta-analyses that directly compared LIT versus HIT protocols on VO_2_max was, ostensibly, reported as either trivial or inconclusive. Three out of the six included meta-analyses reported small/moderate beneficial effects of HIT over LIT (*α* < 0.05) [20, 23, 27]. However, two of these reviews reported “substantial” heterogeneity (*I*^*2*^>0.75) [28], small-study bias (*p* < 0.10) [29], a relatively small pooled sample size (i.e., <1,000 participants), had a high degree of overlap (CCA = 11%) and reported several moderators (e.g., baseline fitness levels, age, HIT variables [e.g., volume, frequency, and duration]), which likely affected results. All three reviews identified that, compared to LIT, HIT generally elicited a greater increase in VO_2_max in older and less fit populations, and/or when long-interval (2 to 4 minutes of work/bout), high-volume (15-minute work/session), and moderate/long-duration HIT protocols (>4 weeks) were prescribed [20, 23, 27]. Furthermore, several primary studies within these reviews concluded that HIT had greater beneficial effects for older participants, whereas LIT showed greater effects for participants with lower baseline fitness [19, 21, 24]. Therefore, it is difficult to conduct a crude comparisons between LIT and HIT, where a more nuanced approach is required (i.e., not all populations respond the same nor are all HIT protocols consistent, therefore giving disparate responses because of other training parameters, rather than intensity per se).

### 4.2. Systematic Review Findings

In healthy older participants, Montero et al. [21] reported an increase in VO_2_max following a LIT intervention (SMD = 0.79, CI = 0.41 to 1.17) with the HIT intervention prescribed at 60–80% VO_2_max also eliciting an improvement in VO_2_max (SMD = 0.95, CI = 0.64 to 1.25). Analysis revealed that none of the assessed potential cofactors (i.e. gender, training characteristics, and methodological quality) moderated the improvement in VO_2_max. Furthermore, Montero et al. [24] reported similar results in a younger population but found that training interventions using cycling (ergometer) showed a greater increase in VO_2_max compared with studies that undertook LIT running (treadmill) (SMD = 1.06 vs. 0.43). As expected, Huang et al. [19] showed that older sedentary individuals who performed LIT (55–60% VO_2_max) for 30–35 minutes per session, three times per week for 16–20 weeks, could improve their VO_2_max by approximately 3.8 mL kg.min^−1^ (∼16% improvement). Moreover, recent “big data” research, on real-world running activities of ∼14,000 individuals with ∼1.6 million exercise sessions and a total distance of ∼20 million km, found that faster runners partake in greater volumes of LIT than slower runners, which was associated with better performance during high-intensity exercise [30].

Research has shown that HIT increases VO_2_max in healthy adults [24]. Scribbans et al. [22] found that HIT (80–92.5% VO_2_max) was a powerful method for eliciting improvements in VO_2_max (0.26 ± 0.10 L.min^−1^, ES = 0.68). Weston et al. [14] included a cohort of both healthy and sedentary participants, reporting moderate improvements in VO_2_max for both active nonathletic (6.2 ± 3.1%) and sedentary men (10 ± 5.1%), as well as active nonathletic (3.6 ± 4.3%) and sedentary women (7.3 ± 4.8%), when compared to a control group (1.2 ± 2.0%). Wen et al. [20] found that the degree of change in VO_2_max induced by HIT varied by population, with greater improvements seen from a healthy nonathletic population, compared to an athletic population. Although all HIT protocols only evoked a small/moderate increase in VO_2_max over LIT programmes [27], long-interval (≥2 min) (SMD = 1.07 (CI = 0.62, 1.52)), high-volume (≥15 min) (SMD = 1.04 (CI = 0.54, 1.54)), and moderate-to-long duration (≥4–12 weeks) (SMD = 0.77 (CI = −0.08, 1.61)) HIT programmes evoked a greater increase in VO_2_max [20], which highlights that a more nuanced approach is required in view of other training variables alongside intensity during program design. Finally, Sloth et al. found that 2–8 weeks of HIT, performed 2–3 times a week, showed VO_2_max improvements (SMD = 0.63, CI = 0.39 to 0.87) for both sedentary and healthy participants [15]. However, when compared to LIT, Gist et al. [26] found small effects between HIT and LIT (SMD = 0.04, CI = -0.17 to 0.24). These findings provide evidence in support of HIT but as their meta-analysis concluded, the difference in the increase between HIT and LIT was either trivial or inconclusive in healthy participants.

### 4.3. Limitations and Future Research Considerations

It is evident that both LIT and HIT modalities carry their own limitations. Scribbans et al. [22] noted that a lack of an exercise intensity effect was specifically related to LIT interventions during short-term studies that were not comparable to HIT interventions. Therefore, work-matched (i.e., per session and over the total training period) training loads are required to make accurate comparisons between HIT and LIT interventions [22]. And the use of training impulse (TRIMP) (i.e., usually the product of training intensity and time) may provide greater insight into the relative efficacy of LIT and HIT interventions [30]. This also highlights the need for standardisation of training with large variation in control of the independent variable (i.e., intensity) reported between studies. It was noted that several of the reviews in this overview prescribed HIT interventions that could have been (re)classified as LIT [21, 24]. Furthermore, with reference to training intensity, standardisation is required for some of the other training variables that are (sometimes inextricably) linked to training intensity (e.g., frequency, volume, and duration). Gist et al. [26] stated that the duration of training interventions in most SIT studies was <6 weeks, questioning the long-term improvements and adaptations within these studies.

Broader limitations exist around sample population, study design, and sample size. Whilst some reviews reported heterogeneity among their included studies [20], others only included studies of young healthy sedentary or recreationally active adults [14, 15, 22] or older populations [19, 21]. As noted previously, there are a greater number of males across the 11 included reviews. Removing any potential bias through RCTs is an important consideration. Scribbans et al. [22] reported that none of their included studies applied RCTs, Sloth et al. [15] reported only four studies that applied RCTs design, and Gist et al. [26] reported that the majority of included studies were RCTs. Finally, small/underpowered sample sizes are a re-occurring problem, as recruitment, adherence, and commitment are difficult. Montero et al. [21] stated that the majority of findings were derived from a relatively small number of studies reporting a lack of statistical power [15] and potential publication bias in sample sizes of between eight and ten participants, with the aggregation of data suggesting publication bias is likely [20]. Therefore, our overview begins to overcome these issues and highlights the need for carefully controlled research designs.

Other specific considerations need to be considered such as outdated technology, and possibly, how less reliable methods for measuring VO_2_max might affect the validity and reliability of results from early studies [19]. HIT warrants high levels of motivation and this may present as an issue to long-term adherence [14]. Finally, the extraction of relative values (ml.kg.min^−1^) rather than absolute values (L.min^−1^) of VO_2_max may magnify the training effect due to a possible decrease of body mass during the training intervention [20]. These considerations should provide future researchers with some guidance around the interpretation of previously published research and future aggregation of these findings.

## 5. Perspective

The findings of this review show that both LIT and HIT are effective at improving VO_2_max and overlap analysis between reviews highlights the need for such an approach to synthesising the literature. Evidence from meta-analyses that compared LIT versus HIT protocols on VO_2_max reported either small, trivial, or inconclusive effects between training groups. Three reviews reported that HIT has potentially greater beneficial effects for older and/or less fit participants versus LIT. Interestingly, smaller effects were seen for longer HIT intervals and may suggest the importance of exercise intensity even between different HIT modalities (e.g., SIT and HIIT). Finally, several methodological considerations are highlighted in this overview, such as the sample population, research design, sample size, and intervention duration. Other specific considerations include technology used to control, monitor, and administer the exercise intensity, control of other (extraneous) training parameters (e.g., work, TRIMP), to allow accurate comparisons to be made different exercise intensities, and standardised nomenclature around training intensity guidelines and categorisation in research and practice.

## Figures and Tables

**Table 1:** Search strategy: key words used for the literature search.

Set		Search Terms
#1		High-intensity interval training OR high-intensity intermittent training OR sprint interval training OR endurance training OR continuous endurance training OR aerobic training OR maximal oxygen uptake OR peak oxygen uptake OR VO_2_max OR moderate intensity continuous training
#2	*AND*	Cardiometabolic OR cardiovascular OR cardiorespiratory
#3	*AND*	Review of literature OR literature review OR meta-analysis OR systematic review
#4	*NOT*	Animals OR masters OR paraplegic OR injury OR disease OR obese OR overweight OR altitude OR cross-sectional study OR obesity OR children OR adolescents OR teenagers OR physical activity OR heat

**Table 2:** Summary of reviews included within the overview, participant, and background information.

Author	Type of review	AMSTAR	Number of studies	Training group	Sample size	Sex	Age (years)	Training status
*MICT*								
Huang et al. [19]	Meta-analysis	7	41	MICT = 50 CON = 43	MICT = 1257 CON = 845	Not reported	MICT = 67.1 ± 4.7 CON = 67.7 ± 5.4	Sedentary

*HIIT*								
Wen et al. [20]	Meta-analysis	9	35	HIIT = 29 MICT = 18 EX-CON = 9 CON = 15	HIIT = 433 MICT = 207 EX-CON = 68 CON = 218	M = 687 F = 276	Range: 19.4–43.1 Mean: 24.3 ± 4.7	Healthy athletic
Montero et al. [21]	Systematic review meta-analysis	7	14	HIIT = 9 MICT = 15	Total = 153	M = 115 F = 38	Range: 42–71 Mean: 61.72 ± 7.58	Healthy
Scribbans et al. [22]	Metaregression meta-analysis	7	28	HIIT = 28, CON = 12	T1 = 136 T2 = 134 T3 = 120	M = 95, F = 41. M = 107, F = 27. M = 79, F = 41.	T1 = 23 ± 1 T2 = 23 ± 1 T3 = 22 ± 2	Healthy active
Milanović et al. [23]	Systematic review meta-analysis	8	28	HIIT = 28 MICT = 24	HIIT = 269 MICT = 204 CON = 246	M = 455 F = 194 Mix = 70	Range: 18–50.3 Mean: 25.1 ± 5	Healthy, untrained, sedentary, recreational
Montero et al. [24]	Systematic review meta-analysis	10	9	HIIT = 4 MICT = 6	Total = 130	M = 120 F = 10	Range: 22–28	Healthy
Weston et al. [14]	Meta-analysis	9	32	HIIT = 36 MICT = 19 EX-CON = 11	HIIT = 343 MICT = 69 EX-CON = 95	HIIT: M = 251, F = 92. END: M = 36, F = 33. EX-CON: M = 62, F = 33.	HIIT:3.62 ± 3.31 MICT: 22.43 ± 2.14 EX-CON: 4.62 ± 4.41	Sedentary active
Bacon et al. [25]	Meta-analysis	6	36	HIIT and MICT: not reported	Total = 334	M = 214, F = 120	Range: 18–42	Healthy

*SIT*								
Gist et al. [26]	Systematic review meta-analysis	8	16	SIT = 16 EX-CON = 16	SIT = 179 EX-CON = 139	M = 97 F = 75 Mix = 146	Mean: 23.5 ± 4.3	Healthy sedentary Trained recreational
Sloth et al. [15]	Systematic review meta-analysis	7	13	SIT = 19, MICT = 13	SIT = 190, MICT = 262	SIT: M = 121, F = 69. END: M = 181, F = 81.	Not reported	Healthy overweight

*HIIT and SIT*								
Maturana et al. [27]	Meta-analysis	8	21	HIIT = 11 SIT = 15 MICT = 25	HIIT = 144 SIT = 149 MICT = 270	M = 343 F = 44	Range: 20–64 Mean: 29.1 ± 12	Sedentary active

HIIT: high-intensity interval training; CON: nonexercise control; MICT: moderate-intensity continuous training; EX-CON: exercising control; SIT: sprint interval training; M: male; F: female; mix: male and female; T1: 60–70% VO2max; T2: 80–92.5% VO2max; and T3: 100–250% VO_2_max.

**Table 3:** Summary of LIT and HIT training interventions included within each review, training prescription, and evaluation.

Author	Mode	Duration (weeks)	Frequency (times per week)	Intensity (as reported in reviews)	VO_2_max
*MICT*					
Huang et al. [19]	Walking (80%), jogging, cycling, stair-climbing, aerobic dance, tai chi, outdoor and aerobic games.	38.1 ± 10	Total = 3.3 ± 0.7	MICT: HRmax (*n* = 19, 60–85% (73.3 ± 6.2%)), VO_2_max (*n* = 10, 50–82% (63.5 ± 10.4%)), HRR (*n* = 28, 35–80% (62.0 ± 13.1%)), HRmax (*n* = 10, 107–129 bpm 119.8 ± 7.5 bpm))	MICT: (mean ± SEM, 3.50 ± 0.84 mL.kg.min^−1^; 95% CI: 1.83–5.17; *p* < 0.001), CON: (0.27 ± 0.91 mL.kg.min^−1^; 95% CI: –2.08 to 1.54; *p*=0.769 )

*HIIT*					
Wen et al. [20]	Cycling (*n* = 21) Handcycling (*n* = 1) Running (*n* = 10) Walking (*n* = 1) Swimming (*n* = 1) Rowing (*n* = 2)	6.62 ± 3.46	Total = 3.17 ± 0.94	HIIT: vVO_2_max (*n* = 3, 100–110%), All out (*n* = 11), pVO_2_max (n = 3, 100–125%), VO_2_max (*n* = 6, 80–120%), VO_2_peak (*n* = 1, 90%), Wmax (*n* = 2, 80-90%), LT (*n* = 1, 120–140%), HRR (*n* = 2, 80-90%), HRmax (*n* = 3, 85–97.5%), maximal effort (*n* = 2), near maximal (*n* = 1), PPO (*n* = 1, 175%). MICT: GET (*n* = 1, 90%), VO_2_max (*n* = 6, 60–70%), VO_2_peak (*n* = 3, 65%), LT (*n* = 1, 80-95%), HRR (*n* = 2, 50–55%), HRmax (*n* = 4, 65–80%), pVO_2_wmax (*n* = 1, 65%). EX-CON: 13 km/hr (*n* = 1), HRmax (*n* = 1, 70%), VO_2_max (*n* = 1, 80%), vVO_2_peak (*n* = 1, 50%)	Healthy: HIIT vs. CON: large effect (SMD = 5.45 mL.kg.min^−1^; SMD = 1.81, 95% CI 1.39–2.22, *p* < 0.05). HIIT vs. MICT: moderate effect (SMD = 2.06 mL.kg.min^−1^; SMD = 0.64, 95% CI: 0.23–1.05, *p* < 0.05). Athletic: HIIT vs. CON: small effect (SMD = 1.71 mL.kg.min^−1^; SMD = 0.57, 95% CI 0.13–1.01, *p* < 0.05)

Montero et al. [21]	Walking (*n* = 7) Running (*n* = 7) Cycling (*n* = 13) Rowing (*n* = 4)	23.95 ± 17.85	Total = 2.56 ± 0.88	HIIT: HRmax (*n* = 9, 70–90%), VO_2_max (*n* = 5, 60–80%), Wmax (*n* = 2, 90–100 %), HRR (*n* = 1, 80%). MICT: not reported	MICT vs. HIIT: SMD: 0.95 (95% CI: 0.64, 1.25), *p* < 0.0001. MICT: SMD: 0.79 (95% CI: 0.41, 1.17), *p* < 0.0001

Scribbans et al. [22]	Cycle (*n* = 26) Running (*n* = 13) Ball dribbling (*n* = 1) Snowshoeing (*n* = 1)	T1 = 6 ± 0.3 T2 = 6.9 ± 0.4 T3 = 6.2 ± 0.3	T1 = 3.7 ± 0.3 T2 = 3.2 ± 0.2 T3 = 3.1 ± 0.2	MICT: T1 = 68(60–70% VO_2_max). HIIT: T2 = 87(80–92.5% VO_2_max), T3 = 167(100–250% VO_2_max)	T1: Pre = 3.2 ± 0.2 (L.min^−1^), Post = 3.5 ± 0.2 (0.29 ± 0.15, ES = 0.77). T2: Pre = 3.8 ± 0.2, Post = 4.1 ± 0.2 (0.26 ± 0.10, ES = 0.68). T3: Pre = 3.2 ± 0.2, Post = 3.5 ± 0.2 (0.35 ± 0.17, ES = 0.80)
Milanović et al. [23]	Not reported	HIIT = 8.86 ± 5.01 END = 9.62 ± 5.43	HIIT = 3.2 ± 2.98 END = 3.32 ± 2.87	HIIT: all out (*n* = 6), HRmax (*n* = 4, 90–100%), HRR (*n* = 1, 100%), VO_2_max (*n* = 7, 80–170%), Pmax (*n* = 1, 125%), pVO_2_max (*n* = 1, 80%), MAS (*n* = 1, 105–110%), vVO_2_max (*n* = 2, 75–130%), pVO_2_max (*n* = 1, 80%), WRmax (*n* = 1, 120%), LT (*n* = 1, 120–140%). MICT: HRmax (*n* = 6, 60-80%), HRR (*n* = 2, 75–85%), VO_2_max (*n* = 9, 60–70%), VO2peak (*n* = 4, 65%), vVO_2_max (*n* = 1, 75%), VLT (*n* = 1, 75–85%), LT (*n* = 1, 80–85%)	HIIT: 5.5 ± 1.2 mL.kg.min^−1^. MICT: 4.9 ± 1.4 2 mL.kg.min^−1^

Montero et al. [24]	Cycle ergometer (*n* = 7) Treadmill (*n* = 6)	5–12.9	Total = 1.17-4.41	HIIT: Wmax (*n* = 1, 50–75%), HRmax (*n* = 7, 70–100%), VO_2_max (*n* = 5, 60–95%). MICT: not reported	MICT: SMD: 0.64, 95% CI: 0.23–1.05, *p*=0.002. HIIT: SMD: 0.87 (CI: 0.22–1.51), *p*=0.008

Weston et al. [14]	Wingate cycling	HIIT = 5.3 ± 2.25 END = 4.9 ± 2.1 EX-CON = 4.4 ± 2.7	HIIT = 2.8 ± 0.5 MICT = 3.4 ± 1.1 EX-CON = 4	HIIT: VO_2_max (*n* = 3, 95–130%), Vmax (*n* = 1, 93%), Pmax (*n* = 8, 90–175%), all out (*n* = 23). MICT: VO_2_max (*n* = 5, 65–80%), GET (*n* = 1, 90%), Pmax (*n* = 1, 60–70%), HRmax (*n* = 1, 70–80%). EX-CON: vVO_2_max (*n* = 1, 75%)	HIIT: 6.2%, ±3.1 (90% CI). MICT vs. HIIT: −1.6% ±4.3. EX-CON: 1.2% ±2.0

Bacon et al. [25]	Running Cycling	6–12	Not reported	HIIT: not reported MICT: 30–48 min/day at 64.3 ± 3.7% VO2max	HIIT: Random effects model: 0.51 L.min^−1^ (95% CI: 0.43–0.60, *p*=0.001 ). SMD = 0.86 (95% CI: 0.72 to 0.99). MICT: not reported

*SIT*					
Gist et al. [26]	Cycling (*n* = 10) Running (*n* = 7)	4.8 ± 2.3	Total = 2.9 ± 0.4	SIT: All out (*n* = 13), Maximal (*n* = 1), PPO (*n* = 1, 175%), VO_2_max (*n* = 1, 130%). EX-CON: VO2max (*n* = 6, 65-80%), HRmax (*n* = 1, 70–80%), GET (*n* = 1, 90%), NA (*n* = 7), Moderate (*n* = 1), Low-moderate (*n* = 1)	SIT: -2.43-11.84% (Cohen's *d* = 0.32, 95% CI : 0.10–0.55; *z* = 2.79, *p* < 0.01). SIT vs. MICT: 2.17–13.49% (Cohen's *d* = 0.04, 95% CI : −0.17 to 0.24; *z* = 0.36, *p*=0.72 ). SIT vs. EX-CON: Cohen's *d* = 0.69, 95% CI: 0.46–0.93; *z* = 5.84, *p* < 0.01)
Sloth et al. [15]	Wingate tests (*n* = 18) Treadmill (*n* = 1)	4.46 ± 2.3	Total = 3	SIT: all out: 30 seconds (*n* = 9), 10–15 seconds (*n* = 3). MICT: not reported	SIT: Range: 4.2–13.4%. Mean: 8.54 ± 3.05%. SMD: 0.63, 95% CI: (0.39–0.87)

*HIIT and SIT*					
Maturana et al. [27]	Cycling (*n* = 20) running (*n* = 5)	8.76 ± 9.03	HIIT = 3.08 ± 0.28 SIT = 3.07 ± 0.27 MICT = 3.32 ± 0.69	HIIT: VO_2_max (*n* = 6, 75–101%), PPO (*n* = 1, 100%), ∆LT (*n* = 1, 35–75%). SIT: maximal at resistance % BM (*n* = 9, 7.5%), %BM (*n* = 2, 5%), VO_2_max (*n* = 2, 100–170%), HRmax (*n* = 1, 90–95%) MICT: VO_2_max (*n* = 17, 50–70%), LT (*n* = 2, 90–95%), HRR (n = 2, 50–60%), HRmax (*n* = 3, 64–80%)	HIIE vs. MICT: small effect (SMD = 0.25, 95% CI: 0.04–0.48, *p*=0.022)

HIIT: high-intensity interval training; CON: nonexercise control; MICT: moderate-intensity continuous training; EX-CON: exercising control; SIT: sprint interval training; VO_2_max: maximal oxygen uptake; T1: 60–70% VO_2_max; T2: 80–92.5% VO_2_max; T3: 100–250% VO_2_max; HRmax: maximal heart rate; HRR: heart rate reserve; Vmax: maximal velocity at VO_2_max; vVO2max : velocity at VO_2_max; Pmax: peak watt work load; GET: gas exchange threshold; pVO_2_max: maximal aerobic power; MAS: maximal aerobic speed; WRmax: work rate at VO_2_max; ∆LT: change between lactate threshold VO2max; VO_2_peak: peak oxygen uptake; VLT: velocity at lactate threshold; Wmax: maximal power output; PPO: peak power output; maximal at resistance of %BM: 30 second all-out effort relative to % body mass; and HIIE: combination of HIIT and SIT training effects on VO_2_max
